# Delaware Dental Law

**Published:** 1885-08

**Authors:** 


### DENTAL LAW.

### Delaware Dental Law.

The following is the text of an act in relation to the practice of dentistry in the State of Delaware, passed by the Legislature, at Dover, March 31, 1885:

*Be it enacted by the Senate and House of Representatives of the State of Delaware in General Assembly met:*

SECTION 1. That it shall be unlawful for any person who is not, at the time of the passage of this act, a recognized practitioner of dentistry in this State, and so recognized by the profession, to practice dentistry, unless he or she shall have obtained a certificate as hereinafter provided, or shall hold a diploma from a reputable dental college, and so recognized by the board of examiners herein created.

SEC. 2. That a board of examiners, to consist of five reputable practicing dentists, is hereby created, whose duty it shall be to carry out the purposes and enforce the provisions of this act; the members of said board shall be appointed by the Governor, who shall select them from the dentists residing in the State. The term for which the members of said board shall hold their offices shall be four years, except that two members of the board first to be appointed under this act, shall be designated by the Governor to hold their offices for the term of two and three and four years, respectively, unless sooner removed by the Governor, and until their successors shall be duly appointed; in a case of vacancy occurring in such board, such vacancy shall be filled in like manner by the Governor.

SEC. 3. That said board shall choose one of it members president and one secretary thereof; it shall fix the time and place of its meeting or meetings; a majority of said board shall at all times constitute a quorum, and the proceedings thereof shall at all reasonable times be open to public inspection; the board shall also make an annual report of its proceedings to the Governor.

SEC. 4. That within six months from the time this act takes effect it shall be the duty of every person who is at that time engaged in the practice of dentistry in this State to cause his or her name and residence or place of business to be registered with said board of examiners, who shall keep a book for that purpose; the statement of every such person shall be verified under oath before a notary public or justice of the peace, in such a manner as may be prescribed by the said board of examiners; every person who shall so register with said board as a practitioner of dentistry may continue to practice the same as such, and shall receive a certificate of such registration upon his or her paying the said board one dollar for such certificate.

SEC. 5 That any and all persons who shall desire to commence such practice "after the passage of this act" shall appear before said board at any of its regular meetings, and be examined with reference to their knowledge and skill in dental surgery, and if the examination of any such person or persons shall prove satisfactory to said board, the board of examiners shall issue to such persons as they shall find to possess the requisite qualifications a certificate to that effect, in accordance with the provisions of this act, upon the payment of one dollar for such certificate; all certificates issued by said board shall be signed by its officers, and such certificates and diplomas, granted as aforesaid, shall be *prima facie* evidence of the right of the holder to practice dentistry in the State of Delaware.

SEC. 6. That any person who shall wilfully violate any of the provisions of this act shall be deemed guilty of a misdemeanor, and upon conviction thereof, in any court having criminal jurisdiction, may be fined not less than fifty dollars nor more than three hundred, or be confined not more than six months in the county jail, in the discretion of the court; all fines received under this act shall be paid into the common school fund of the city or county in which such conviction takes place.

SEC. 7. That the board of examiners shall meet within thirty days after appointment, and frame by-laws governing the board, and any person or persons desiring to be examined by the board of examiners for a certificate to practice dentistry in this State shall give notice of such desire to the secretary of said board, who shall notify the members thereof, and they shall, within fifteen (15) days from the receipt of such notice, meet to examine such person or persons, and give him, her, or them proper notice of such meeting.

SEC. 8. That nothing in this act shall be so construed as to interfere with the rights and privileges of physicians and surgeons in the discharge of their professional duties.

SEC. 9. That this act shall take affect from the date of its passage.

Governor Stockley, on the 25th of April, appointed the following to constitute the Board of Examiners: Drs. T. H. Gilpin, Middletown; C. R. Jeffries, Wilmington; C. H. S. Littleton, Milford; E. Lewis, Dover, and C. Porter.

